# Modeling CaMKII-mediated regulation of L-type Ca^2+^ channels and ryanodine receptors in the heart

**DOI:** 10.3389/fphar.2014.00060

**Published:** 2014-04-03

**Authors:** Joseph L. Greenstein, Panagiota T. Foteinou, Yasmin L. Hashambhoy-Ramsay, Raimond L. Winslow

**Affiliations:** Institute for Computational Medicine, Department of Biomedical Engineering, Johns Hopkins UniversityBaltimore, MD, USA

**Keywords:** cardiac myocyte, excitation-contraction coupling, cell signaling, CaMKII, computational modeling

## Abstract

Excitation-contraction coupling (ECC) in the cardiac myocyte is mediated by a number of highly integrated mechanisms of intracellular Ca^2+^ transport. Voltage- and Ca^2+^-dependent L-type Ca^2+^ channels (LCCs) allow for Ca^2+^ entry into the myocyte, which then binds to nearby ryanodine receptors (RyRs) and triggers Ca^2+^ release from the sarcoplasmic reticulum in a process known as Ca^2+^-induced Ca^2+^ release. The highly coordinated Ca^2+^-mediated interaction between LCCs and RyRs is further regulated by the cardiac isoform of the Ca^2+^/calmodulin-dependent protein kinase (CaMKII). Because CaMKII targets and modulates the function of many ECC proteins, elucidation of its role in ECC and integrative cellular function is challenging and much insight has been gained through the use of detailed computational models. Multiscale models that can both reconstruct the detailed nature of local signaling events within the cardiac dyad and predict their functional consequences at the level of the whole cell have played an important role in advancing our understanding of CaMKII function in ECC. Here, we review experimentally based models of CaMKII function with a focus on LCC and RyR regulation, and the mechanistic insights that have been gained through their application.

## Introduction

Cardiac electrophysiology is a discipline with a rich and deep history dating back more than a half-century. Much of our understanding of fundamental biological mechanisms in this field comes from experimental research coupled with integrative mathematical modeling. The goal of this modeling has been to achieve a quantitative understanding of the functional mechanisms and relationships that span from the level of molecular structure and function to integrated cardiac myocyte behavior in health and disease. Advances in computational techniques and hardware have led to the recent development of many sophisticated large-scale models of heart tissue, in which these electrophysiological myocyte models are the fundamental building blocks. Some of the most fundamental advances in computational cell biology, including formulation of dynamic models of voltage-gated ion channels, mitochondrial energy production, membrane transporters, intracellular Ca^2+^ dynamics, and signal transduction pathways have emerged from this field. One such signaling pathway is that involving the Ca^2+^/calmodulin (CaM)-dependent protein kinase (CaMKII), which targets and functionally regulates a number of proteins that are central to cardiac excitation-contraction coupling (ECC), and has been identified as a promising new target for antiarrhythmic therapy in patients with heart failure (Rokita and Anderson, 2012). This review focuses on the role of experimentally based models of CaMKII function with a focus on L-type Ca^2+^ channel (LCC) and ryanodine receptor (RyR) regulation, and the mechanistic insights that have been gained through their application.

The nature of ECC is linked closely to the micro-anatomical structure of the cell. Sarcomeres, the basic units of contractile proteins, are bounded on both ends by the t-tubular system (Soeller and Cannell, 1999; Bers, 2001). The t-tubules extend deep into the cell and approach the sarcoplasmic reticulum (SR), an intracellular luminal organelle involved in the uptake, sequestration and release of Ca^2+^. The junctional SR (JSR) is the portion of the SR most closely approximating (within 12–15 nm Franzini-Armstrong et al., 1999) the t-tubules. The close proximity of these two structures forms a microdomain known as the dyad. RyRs are Ca^2+^-sensitive Ca^2+^-release channels which are preferentially located in the dyadic region of the JSR membrane. In addition, LCCs are preferentially located within the dyadic region of the t-tubules, where they are in close opposition to the RyRs. The process by which Ca^2+^ enters the myocyte during the initial depolarization stages of the action potential (AP) via voltage-gated LCCs and triggers the opening of RyRs, and hence JSR Ca^2+^ release, is known as Ca^2+^-induced Ca^2+^ release (CICR) and comprises the initial transduction event of ECC. Recent imaging studies have demonstrated that dyadic clefts are small, where junctions contain an average of 14 RyRs, with some clusters incompletely filled with RyRs, and with a large fraction of clusters closely spaced within 20–50 nm, suggesting that smaller clusters may act together to function as a single site of CICR (Baddeley et al., 2009; Hayashi et al., 2009).

The elucidation of CICR mechanisms became possible with the development of experimental techniques for simultaneous measurement of L-type Ca^2+^ current (I_CaL_) and Ca^2+^ transients, and detection of local Ca^2+^ release events known as Ca^2+^ sparks (Cheng et al., 1993). The evidence for tight regulation of SR Ca^2+^ release by triggering LCC current (for a review see Soeller and Cannell, 2004) gave rise to and later verified the local control theory of ECC (Stern, 1992). The mechanism of local control predicts that tight regulation of CICR is achieved because LCCs and RyRs are regulated by local dyadic Ca^2+^. It is now widely accepted that CaMKII, a ubiquitous Ca^2+^-dependent protein that can become highly activated in the dyad (Currie et al., 2004; Hudmon et al., 2005; Maier, 2005), can modulate CICR via phosphorylation of a number of ECC proteins including LCCs and RyRs (Maier and Bers, 2007). CaMKII modulates LCCs via a Ca^2+^-dependent positive-feedback regulatory mechanism known as I_CaL_ facilitation (Anderson et al., 1994; Xiao et al., 1994; Yuan and Bers, 1994). This is observed as a positive staircase in current amplitude in combination with a slower rate of I_CaL_ inactivation upon repeated membrane depolarization. Although multiple mechanisms have been suggested to underlie I_CaL_ facilitation, experiments from Dzhura et al. (2000) demonstrated that CaMKII-dependent I_CaL_ facilitation alters the behavior of LCCs such that high activity gating modes with prolonged open times are more likely to occur. The specific functional effects of CaMKII phosphorylation of RyRs remain controversial. CaMKII has been shown to increase (e.g., Wehrens et al., 2004) or decrease (e.g., Lokuta et al., 1995) the channel open probability. Inconsistencies in experimental findings regarding the role of CaMKII on RyR function may not be surprising given the methodological differences between the studies. In addition, the high sensitivity of RyR gating to both cytosolic and SR Ca^2+^ makes it difficult to interpret experiments in which these Ca^2+^ concentrations are not tightly controlled. In one well designed study, Guo et al. (2006) developed an experimental protocol that overcame this challenge and provided strong evidence that CaMKII mediated phosphorylation of RyRs increases channel open probability. Such an increase is also consistent with transgenic studies of CaMKII overexpression which report increased Ca^2+^ spark frequency in response to elevated CaMKII activity (Maier et al., 2003; Kohlhaas et al., 2006).

The CaMKII holoenzyme exists as an elaborate macromolecular complex consisting of two stacked ring-shaped hexamers (Hudmon and Schulman, 2002). Each of the holoenzyme's 12 subunits can be activated through the binding of Ca^2+^-bound CaM (Ca^2+^/CaM) to the CaMKII regulatory domain in response to beat-to-beat transient increases of intracellular Ca^2+^ concentration. In cardiac myocytes, an activated CaMKII molecule can undergo autophosphorylation by neighboring subunits at threonine amino acid residues in its regulatory domain, which allows the kinase to retain activity even upon dissociation of Ca^2+^/CaM (Meyer et al., 1992). An alternative mechanism of oxidative CaMKII activation has also been identified (Erickson et al., 2008). As a result of the fact that CaMKII regulates multiple protein targets (both directly and indirectly involved in ECC), and that the effect of CaMKII mediated phosphorylation on any particular target protein may involve complex alterations in biophysical function (e.g., mode-switching behavior of LCCs), the task of elucidating its role in cardiac function in both normal and failing hearts continues to present great challenges and cannot be accomplished via experiments alone. Some recent advances in understanding the mechanisms of CaMKII-dependent function in cardiac myocytes have been discovered by coupling mathematical models to experimental observations. In this review we will focus on key models of CaMKII-mediated regulation of LCCs and RyRs arising from both Ca^2+^/CaM-dependent as well as oxidative activation pathways, the integration of such models into whole-cell models, and the mechanistic insights that have been obtained from this work. A summary of the models covered here is provided in Table 1.

**Table 1:** **Key features of CaMKII models in cardiac ECC**.

**Cell model**	**Species**	**Parent ECC model**	**CaMKII activation model**	**Included CaMKII targets**
Hund and Rudy, 2004	Dog	Luo and Rudy, 1994	Hanson et al., 1994	LCCs, RyRs, SERCA/PLB
Iribe et al., 2006	Guinea pig	Noble et al., 1991	Conceptual kinetic model	LCCs, RyRs, SERCA
Livshitz and Rudy, 2007	Guinea pig/Dog	Luo and Rudy, 1994; Hund and Rudy, 2004	Hanson et al., 1994	LCCs, RyRs, SERCA/PLB
Grandi et al., 2007	Rabbit	Shannon et al., 2004	Static formulation	I_CaL_, I_Na_, I_to_
			Target parameters adjusted	
Saucerman and Bers, 2008	Rabbit	Shannon et al., 2004	Adapted from Dupont et al. (2003), Gaertner et al. (2004)	no target
Hund et al., 2008	Dog	Hund and Rudy, 2004	Hanson et al., 1994	LCCs, RyRs, SERCA/PLB, I_Na_
Koivumaki et al., 2009	Mouse	Bondarenko et al., 2004	Bhalla and Iyengar, 1999	LCCs, RyRs, SERCA/PLB
Hashambhoy et al., 2009	Dog	Greenstein and Winslow, 2002	Dupont et al., 2003	LCCs
Christensen et al., 2009	Dog	Hund et al., 2008	Hanson et al., 1994	LCCs, RyRs, SERCA/PLB, I_Na_
Soltis and Saucerman, 2010	Rabbit	Saucerman and Bers, 2008	Dupont et al., 2003; Gaertner et al., 2004	LCCs, RyRs, PLB, I_Na_, I_to_
Hashambhoy et al., 2010	Dog	Hashambhoy et al., 2009	Dupont et al., 2003	LCCs, RyRs, PLB
O'hara et al., 2011	Human	Hund and Rudy, 2004; Decker et al., 2009	Hund and Rudy, 2004	LCCs, PLB, I_Na_, I_to_
Hashambhoy et al., 2011	Dog	Hashambhoy et al., 2010	Dupont et al., 2003	LCCs, RyRs, PLB, I_Na_
Zang et al., 2013	Dog	Hund and Rudy, 2004	Hanson et al., 1994	LCCs, RyRs, I_Na_, I_to_, I_K1_, SERCA
Morotti et al., 2014	Mouse	Soltis and Saucerman, 2010	Dupont et al., 2003; Gaertner et al., 2004	LCCs, RyRs, PLB, I_Na_, I_to_, I_K1_, I_NCX_

## Models of Ca^2+^/CaM-dependent CaMKII activation and regulation

The first model of CaMKII signaling within the context of ECC integrated into the cardiac myocyte was presented by Hund and Rudy (2004). This model, known as the HRd model, incorporates a scheme based on the work of Hanson et al. (1994), where a single population of CaMKII transitions from an inactive to active state in response to elevated subspace Ca^2+^ levels. CaMKII activity is assumed to modify the function of LCCs and RyRs by slowing their inactivation kinetics, thereby enhancing I_CaL_ and JSR Ca^2+^ release, respectively. SR Ca^2+^ uptake was also enhanced by CaMKII activity via its action on phospholamban (PLB) and the SR Ca^2+^-ATPase (SERCA). The model predicted that CaMKII plays a critical role in the rate-dependent increase of the cytosolic Ca^2+^ transient by increasing ECC gain, but that it does not play a significant role in rate-dependent changes of AP duration. Similar results were obtained in the human cardiac AP model of O'hara et al. (2011), which incorporates the role of CaMKII phosphorylation on LCCs, the fast Na^+^ current (I_Na_), and the transient outward K^+^ current (I_to1_). The HRd model was later updated and used to investigate the role of altered CaMKII signaling in the canine infarct border zone (Hund et al., 2008). Experimentally measured elevation of CaMKII autophosphorylation in this region is reproduced by the model, which indicates that hyperactive CaMKII impairs Ca^2+^ homeostasis by increasing Ca^2+^ leak from the SR. A number of additional modeling studies have focused on the role of CaMKII on various aspects of intracellular Ca^2+^ dynamics. Iribe et al. (2006) incorporated a conceptual CaMKII model into their previous myocyte model (Noble et al., 1991) combined with a model of mechanics (Rice et al., 1999) in order to investigate its role on SR Ca^2+^ handling and interval-force relations. They found that a relatively slow time-dependent inactivation of CaMKII allowed for the reconstruction of a variety of interval-force relations, including alternans. Koivumaki et al. (2009) built a model of the murine cardiac myocyte to analyze genetically engineered heart models in which CaMKII-mediated phosphorylation of LCCs is disrupted or CaMKII is overexpressed. They demonstrated how these genetic manipulations lead to the observed experimental phenotypes as a result of autoregulatory mechanisms that are inherent in intracellular Ca^2+^ cycling (e.g., steady-state regulation of SR content via Ca^2+^ release dependent inactivation of LCCs), and that disruption of the regulatory system itself (e.g., via CaMKII overexpression) leads to the most aberrant physiological phenotypes. Livshitz and Rudy (2007) formulated a new model of SR Ca^2+^ release kinetics and incorporated it into the HRd model in order to better understand regulation of Ca^2+^ and electrical alternans under various pacing protocols. They found that increased CaMKII activity leads to increased alternans magnitude as well as the appearance of both Ca^2+^ and electrical alternans at lower pacing rates (where they would not normally occur). This model identifies Ca^2+^ alternans as the underlying mechanism for electrical alternans, both of which were eliminated with CaMKII inhibition, suggesting this as an antiarrhythmic strategy. Zang et al. (2013) recently developed a new canine myocyte model, also based on the HRd model, to study the role of upregulated CaMKII in heart failure. Similarly, they find that enhanced RyR Ca^2+^-sensitivity and SR Ca^2+^ leak mediated by CaMKII overexpression alters Ca^2+^ handling in a manner that promotes alternans, while AP prolongation occurs primarily due to an associated down regulation of K^+^ currents. Interestingly, they find that blocking SR Ca^2+^ leak restores contraction and relaxation function, but does not eliminate alternans completely.

Grandi et al. (2007) developed a model of CaMKII overexpression in the rabbit ventricular myocyte, which incorporates the functional effects of CaMKII-mediated phosphorylation of LCCs, as well as I_Na_ and I_to1_. This model shows that while CaMKII-mediated action on LCCs prolongs the AP, the combined effect of CaMKII on all three of the targets studied leads to AP shortening. While this study was primarily motivated by and focused on the functional role of CaMKII-mediated regulation of I_Na_ (see accompanying article in this series by Grandi and Herren), it clearly demonstrates that the multiple targets of CaMKII interact to produce net alterations of integrated cellular function which are difficult to predict from knowledge if its functional effects on isolated individual targets. Saucerman and Bers (2008) developed and incorporated models of CaM, CaMKII, and calcineurin (CaN) into the rabbit ventricular myocyte model of Shannon et al. (2004) in order to better understand the functional consequences of the different affinities of CaM for CaMKII and CaN during APs. The model predicts that the relatively high Ca^2+^ levels that are achieved in the cardiac dyad lead to a high degree of CaM activity which results in frequency-dependent CaMKII activation and constitutive CaN activation, whereas the lower Ca^2+^ levels in the cytosol only minimally activate CaM, which allows for gradual CaN activation, but no significant activation of CaMKII. The prediction that robust beat-to-beat oscillations of CaMKII activity occur in the dyad (i.e. in the vicinity of RyRs and LCCs) but not in the cytosol is a key factor that would influence the way in which local signaling mechanisms would be incorporated into models that followed that included detailed reconstruction of dyadic Ca^2+^ dynamics. In one such model, Soltis and Saucerman (2010) integrated dynamic CaMKII-dependent regulation of LCCs, RyRs, and PLB with models of cardiac ECC, CaMKII activation, and β-adrenergic activation of protein kinase A (PKA). In this model phosphorylation of all CaMKII substrates exhibits positive frequency dependence similar to that observed in experiments (De Koninck and Schulman, 1998). However, both CaMKII activity and target protein phosphorylation levels adapt to changes in pacing rate with distinct kinetics (see Figure 2 of Soltis and Saucerman, 2010). CaMKII-mediated phosphorylation is relatively fast at LCCs, slower at RyRs, and very slow at PLB. Additionally, the model predicts a high degree of phosphorylation of LCCs in control conditions, but a moderate amount of phosphorylation of RyRs and PLB (e.g., PLB phosphorylation <10% at all frequencies). In addition, this study predicts a novel mechanism in which CaMKII and PKA synergize to form a positive feedback loop of CaMKII-Ca^2+^-CaMKII regeneration. Furthermore, this model predicts that CaMKII-mediated hyperphosphorylation of RyRs, which renders them leaky, may be a proarrhythmic trigger via induction of delayed afterdepolarizations (DADs). Recently, Morotti et al. (2014) modified the Soltis and Saucerman (2010) rabbit model to further investigate the arrhythmogenic role of another synergistic interaction in mouse ventricular myocytes. This synergism is that of the positive feedback loop of CaMKII-Na^+^-Ca^2+^-CaMKII in which CaMKII-dependent increases in intracellular Na^+^ level perturbs Ca^2+^ homeostasis and CaMKII activation. Simulation results from Morotti et al. (2014) demonstrate that the feedback between disrupted Na^+^ fluxes and CaMKII signaling is exaggerated when CaMKII is overexpressed. Under this condition, and upon an increase in intracellular Na^+^ concentration, the model predicts Ca^2+^ overload and enhancement of CaMKII activity which in turn increases spontaneous Ca^2+^ release events via an increase in RyR phosphorylation. This CaMKII-dependent hyperphosphorylation of RyRs exacerbates the associated electrophysiological instability as simulated by the occurrence of DADs (see Figure 7 of Morotti et al., 2014). In this model, the DADs do not occur when CaMKII target phosphorylation is clamped to basal levels.

**Figure 1:**
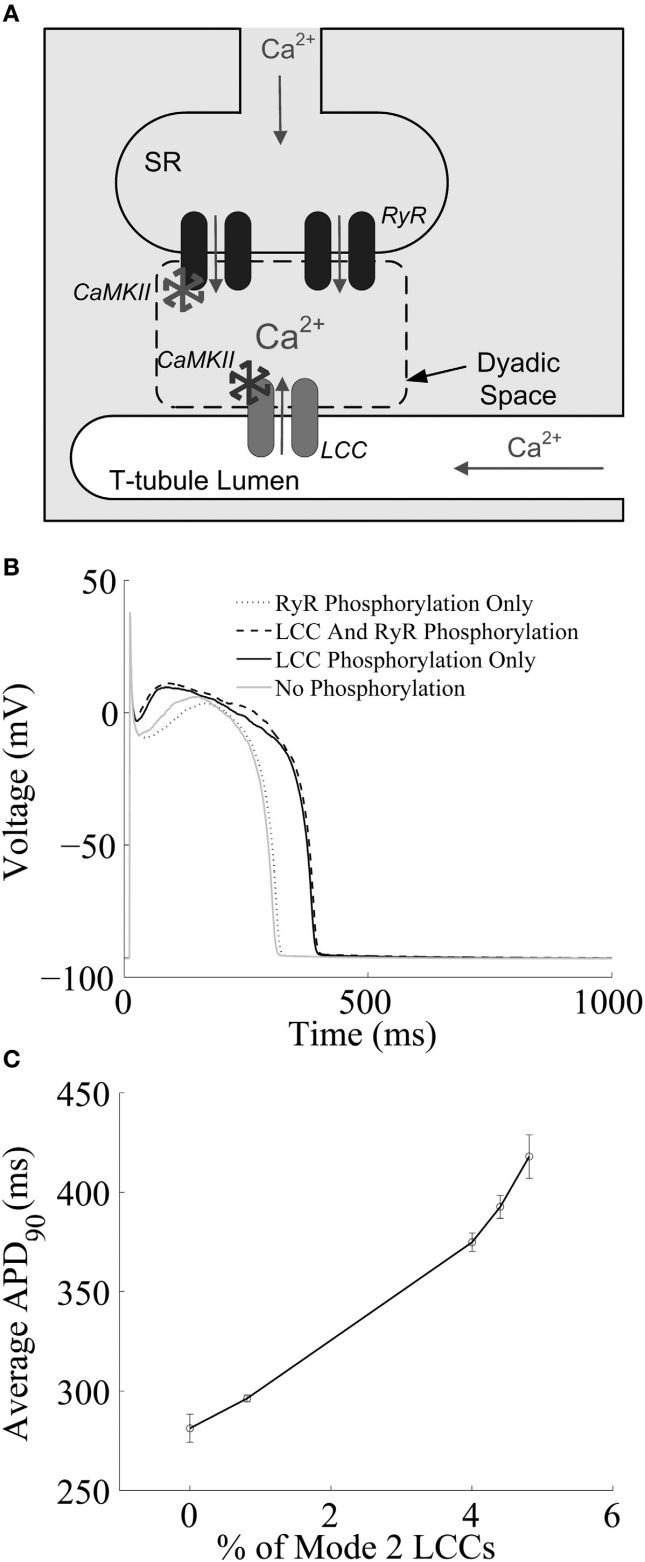
**(A)** Schematic representation of the cardiac dyad. Ca^2+^ ions pass through the LCC and enter the dyad, where they bind to RyRs, triggering CICR. The activity of each CaMKII monomer is a function of dyadic Ca^2+^ levels. Ca^2+^ ions released from the SR diffuse out of the dyad into the bulk myoplasm. **(B)** Simulated results from a 1-Hz AP-pacing protocol under different LCC and/or RyR phosphorylation conditions. **(C)** APD_90_ as a function of average LCC phosphorylation levels. Fully phosphorylated LCCs gate in mode 2, which exibit long-duration openings. **(A)** Reprinted with permission from Hashambhoy et al. (2009). Copyright 2009 Elsevier. **(B,C)** Reprinted with permission from Hashambhoy et al. (2010). Copyright 2010 Elsevier.

Recently, Hashambhoy et al. (2009) developed a biochemically and biophysically detailed model of CaMKII-mediated phosphorylation of LCCs. The model includes descriptions of the dynamic interactions among CaMKII, LCCs, and phosphatases as a function of dyadic Ca^2+^ and CaM levels, and has been incorporated into an integrative model of the canine ventricular myocyte with stochastic simulation of LCC and RyR channel gating within a population of release sites based on the theory of local control of ECC (Greenstein and Winslow, 2002). A schematic representation of one such model Ca^2+^-release site is shown in Figure 1A. This CaMKII-LCC model is formed by the integration of three modules: a CaMKII activity model which reflects the different structural and functional states of the kinase based on the work of Hudmon and Schulman (2002) and Dupont et al. (2003), an LCC phosphorylation model derived from studies of facilitation in LCC mutants (Grueter et al., 2006; Lee et al., 2006), and a previously developed LCC gating model (Jafri et al., 1998; Greenstein and Winslow, 2002). In this model it is assumed that there is one 12-subunit CaMKII holoenzyme tethered to each LCC (Hudmon et al., 2005), each CaMKII monomer can transition among a variety of activity states (see Figure 2 of Hashambhoy et al., 2009), and CaMKII monomers can catalyze phosphorylation of individual LCCs. The LCC gating model reflects two forms of channel gating, mode 1 (normal activity) and mode 2 (high activity with long openings). In the model, LCC phosphorylation promotes transitions of LCCs from mode 1 to mode 2 gating, based on experimental observations with constitutively active CaMKII (Dzhura et al., 2000). This model demonstrates that these CaMKII-dependent shifts of LCC gating patterns into high activity gating modes may be the underlying mechanism of I_CaL_ facilitation. In addition, the model predicts that this CaMKII-mediated shift in LCC gating leads to an apparent increase in the speed of both I_CaL_ inactivation and recovery from inactivation, both of which are experimentally associated with I_CaL_ facilitation, with no change to the underlying intrinsic LCC inactivation kinetics. Hashambhoy et al. (2010) further expanded this model to include CaMKII-dependent regulation of RyRs. In this model update, RyR phosphorylation is modeled as a function of dyadic CaMKII activity and it is assumed that RyR sensitivity to dyadic Ca^2+^ is increased upon phosphorylation, the degree to which is constrained by experimental measurements of Ca^2+^ spark frequency and steady state RyR phosphorylation (Guo et al., 2006). This study demonstrated that under physiological conditions, CaMKII phosphorylation of LCCs ultimately has a greater effect on ECC gain, RyR leak flux, and AP duration as compared with phosphorylation of RyRs (Figure 1B). AP duration at 90% repolarization (APD_90_) correlates well with a CaMKII-mediated shift in modal gating of LCCs (Figure 1C). A modest additional increase in LCC phosphorylation, and hence mode 2 gating, beyond that shown in Figure 1C leads to the appearance of early afterdepolarizations (EADs) in simulated APs (see Figure 5 of Hashambhoy et al., 2010). The results of these model analyses suggest that LCC phosphorylation sites may in fact prove to be a more effective target than those on the RyR for modulating diastolic RyR-mediated Ca^2+^ leak and preventing abnormal proarrhythmic cellular depolarization.

## Models of oxidative CaMKII activation and regulation

The modeling studies described above focus on the role of CaMKII-mediated regulation of its targets via the classical Ca^2+^/CaM-dependent activation pathway which involves CaMKII autophosphorylation. Recently a novel mechanism for oxidative CaMKII activation was discovered in the heart (Erickson et al., 2008). This alternative oxidation-dependent pathway involves the oxidation of CaMKII at specific methionine residues, which has been shown to produce persistent kinase activity and increase the likelihood of arrhythmias (Xie et al., 2009; Wagner et al., 2011). These new findings implicate oxidative CaMKII activation as a putative mechanistic link between the accumulation of reactive oxygen species (ROS) and life-threatening cardiac arrhythmias (Erickson et al., 2011). Therefore, developing a quantitative understanding of the role of CaMKII oxidation in regulating cardiac ECC requires the integration of computational models that link cellular ROS and redox balance, CaMKII activity and function, ECC, and whole-cell electrophysiology. Such models will prove to be powerful tools for teasing out important mechanisms of arrhythmia in heart disease.

In one recent study, Christensen et al. (2009) developed a deterministic model of oxidative CaMKII activation based on their own experimental findings to study the role of this signaling pathway in the canine infarct border zone following myocardial infarction. A new simplified model of CaMKII activation was developed based on the work of Dupont et al. (2003), and incorporated into a model of a single cardiac fiber. Each myocyte within the fiber model was represented by the Hund et al. (2008) model, which as noted above, incorporates CaMKII effects on I_Na_, I_CaL_, SR Ca^2+^ leak and uptake, as well as ion channel remodeling associated with the infarct border zone. Simulation results demonstrate that enhanced oxidative CaMKII activity is associated with reduced conduction velocity, increased refractory periods, and a greater likelihood of conduction block formation. Furthermore, CaMKII inhibition in the model improves conduction and reduces vulnerability to conduction block and reentry. The results of Christensen et al. (2009) are attributed primarily to CaMKII-mediated regulation of I_Na_ kinetics and availability. They note that CaMKII activation will also impact ECC proteins in ways that may promote arrhythmias, but these mechanisms were not explored in detail in this model.

**Figure 2:**
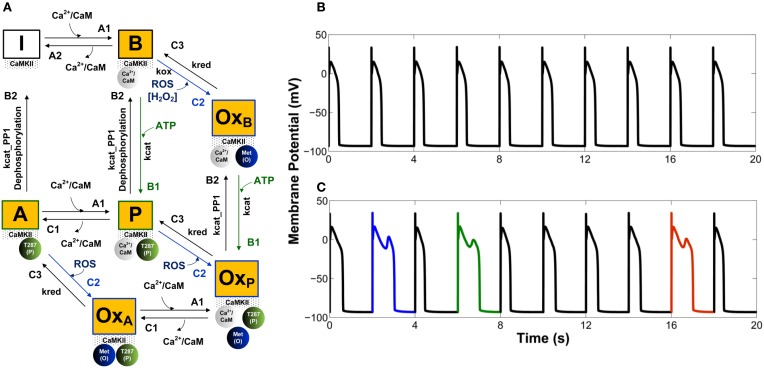
**(A)** State diagram of the stochastic CaMKII activation model of Foteinou et al. (2013). Prior to the introduction of Ca^2+^/CaM, all of the CaMKII subunits are in the inactive form (state I). Activation occurs upon binding of Ca^2+^/CaM (state B), autophosphorylation (state P), and oxidation (state Ox_B_). Autonomous active states (Ca^2+^/CaM-unbound) can be either autophosphorylated (state A) or oxidized (state Ox_A_). The model also includes an active state that is both oxidized and phosphorylated (state Ox_P_). **(B)** Simulated steady state APs under control conditions (0 μM H_2_O_2_). **(C)** Simulated APs, some of which exhibit EADs, under conditions of elevated oxidant stress (200 μM H_2_O_2_). Simulated APs are from a 2 s PCL pacing protocol (ensemble of 12500 calcium release units). For each condition, results for 10 consecutive APs following an initial 10 s of pacing are shown.

Along these lines, Foteinou et al. (2013) have recently developed a novel stochastic model of CaMKII activation (Figure 2A) that reflects the functional properties of the cardiac isoform including both the canonical phosphorylation-dependent activation pathway and the newly identified oxidation-dependent pathway. This model builds upon the work of Hashambhoy et al. (2010, 2011) in order to incorporate recent experimental data for CaM affinity and autophosphorylation/oxidation rates measured specifically for the cardiac isoform of CaMKII (Gaertner et al., 2004; Erickson et al., 2008). Predicated upon this, the authors implemented the four-state deterministic activation model of Chiba et al. (2008) within a stochastic framework that is constrained by the geometry of the CaMKII holoenzyme. This was accomplished by restricting CaMKII autophoshorylation events to occur only between adjacent CaMKII subunits. The stochastic activation model was further modified by including oxidized active states in addition to a Ca^2+^/CaM-bound active state, an autophosphorylated Ca^2+^/CaM-bound state, and an autophosphorylated Ca^2+^/CaM-dissociated state (i.e., an autonomous active state). Following incorporation into the canine cardiac myocyte model (Hashambhoy et al., 2011), Foteinou et al. (2013) obtain steady state APs at a pacing cycle length (PCL) of 2 s as illustrated in Figure 2B. This model predicts increased likelihood of EADs under this protocol upon increased oxidative stress (application of 200 μM H_2_O_2_, which is at the high end of pathophysiological levels), as shown in Figure 2C. Notably, the model simulates no EADs in the presence of ROS at a PCL of 1 s, which is consistent with the experimentally measured rate-dependence of EADs in the presence of 200 μM H_2_O_2_ (Zhao et al., 2012). The model predicts that EADs result from both enhanced CaMKII-dependent shifts of LCCs into highly active mode 2 gating and a progressively increased late component of Na^+^ current (I_NaL_). The simulated increase in I_CaL_ via oxidized CaMKII corroborates the experimental findings of Song et al. (2010) who demonstrated that oxidative CaMKII activation is involved in the regulation of LCCs. In particular, the *in silico* model of H_2_O_2_ exposure discussed here predicts that the fraction of LCCs gaiting in mode 2 will shift from 5% in control (absence of ROS) to 7% upon this increase in ROS. Using this model, the simulated diastolic RyR leak also increases in response to oxidative stress (~44%) primarily as an indirect consequence of increased Ca^2+^ influx via LCCs. Wagner et al. (2011) recently observed a ~15-fold H_2_O_2_-mediated increase in SR Ca^2+^ leak, which is far greater than that produced in this model (<2-fold). They, however, provide additional evidence indicating that their observed increase in Ca^2+^ leak does not require the presence of CaMKII, suggesting an important role for CaMKII-independent mechanisms of ROS-mediated alteration of cardiac ECC as well.

As these ROS-mediated effects need to be further examined, future studies will focus on establishing quantitative links between cellular ROS and redox balance, CaMKII activity and function, ECC, and whole-cell electrophysiology. Recently, Gauthier et al. (2013a,b) developed mechanistic models of ROS production and scavenging to investigate how these two competing processes control ROS levels in cardiac mitochondria. Simulations confirm the hypothesis that mitochondrial Ca^2+^ mismanagement leads to decreased scavenging resources and accounts for ROS overflow as is believed to occur in heart failure (Hill and Singal, 1996). Notably, the ROS regulation module of Gauthier et al. (2013b) enables its use in larger scale heart models designed to simulate and study how mitochondrial ROS and the functional consequences of its accumulation, such as CaMKII oxidation, regulate cellular physiological function, AP properties, and arrhythmogenesis.

## Conclusion

Integrative modeling of cardiac ECC, cell signaling, and myocyte physiology has played a critical role in revealing mechanistic insights across a range of biological scales. With respect to CaMKII function at the smallest scale, models have shed light on how Ca^2+^ ions, CaM, CaMKII, LCCs, and RyRs interact in the cardiac dyadic junction both at rest and during triggered ECC events. On an intermediate scale, models have predicted the consequences of normal and abnormal CaMKII signaling on whole-cell Ca^2+^ cycling, effects of ROS imbalance, AP shape, and the generation of cellular arrhythmias such as EADs. Incorporation of these cellular models into higher scale tissue simulations has provided important insight into the relationship between CaMKII function, electrical wave conduction velocity, and the emergence of arrhythmogenic substrates in diseased tissue. The continuum of biological scales spanned by these models allows for the development of multiscale approaches whereby we can predict and understand the emergence of macroscale phenotypes as a consequence of CaMKII-mediated molecular signaling events. A great deal of evidence now implicates CaMKII as a nexus point linking heart failure and arrhythmias (Swaminathan et al., 2012), identifying it as a prime target for antiarrhythmic therapies (Fischer et al., 2013). Despite this growing body of evidence, the modeling studies presented here demonstrate that it remains difficult to identify which of the many CaMKII target proteins are primarily responsible for the functional changes that increase the likelihood of arrhythmia development. Some studies (Hund et al., 2008; Soltis and Saucerman, 2010; Zang et al., 2013; Morotti et al., 2014) identify phosphorylation of RyRs, which leads to elevated SR Ca^2+^ leak and/or increased likelihood of spontaneous Ca^2+^ release events and DADs, as the key mechanism underlying arrhythmogenesis. Others (Hashambhoy et al., 2010; Foteinou et al., 2013) predict that LCC phosphorylation, which leads to elevated channel open probability via high activity gating, plays the predominant role by altering whole cell Ca^2+^ levels and AP properties, including the appearance of EADs. These mechanisms may co-exist in heart failure, and it is likely that both play an important role. Moreover, CaMKII modulation of other targets such as PLB, I_Na_, and K^+^ currents, many of which have been incorporated into the above cell models, adds additional layers of complexity to the task of interpreting and predicting the mechanisms by which CaMKII signaling alters cardiac function in health and disease. As a result of the breadth and diversity of CaMKII targets, strategies that involve broad inhibition of CaMKII activity will impact and alter the function of many cellular subsystems, with complex consequences that may not all be beneficial. As mathematical models of CaMKII signaling in the cardiac dyad and beyond are further developed, they will play a key role in identifying target-specific therapeutic strategies for improving myocardial function and reducing arrhythmias.

### Conflict of interest statement

The authors declare that the research was conducted in the absence of any commercial or financial relationships that could be construed as a potential conflict of interest.
